# Immersive Virtual Reality as a Novel Physical Therapy Approach for Nonagenarians: Usability and Effects on Balance Outcomes of a Game-Based Exercise Program

**DOI:** 10.3390/jcm11133911

**Published:** 2022-07-05

**Authors:** Pablo Campo-Prieto, José Mª Cancela-Carral, Borja Alsina-Rey, Gustavo Rodríguez-Fuentes

**Affiliations:** 1 Department of Functional Biology and Health Sciences, Faculty of Physiotherapy, University of Vigo, 36005 Pontevedra, Spain; pcampo@uvigo.es (P.C.-P.); gfuentes@uvigo.es (G.R.-F.); 2 Healthyfit Research Group, Galicia Sur Health Research Institute (IIS Galicia Sur), SERGAS-UVIGO, 36213 Vigo, Spain; 3 Department of Special Didactics, Faculty of Education and Sports Science, University of Vigo, 36005 Pontevedra, Spain; borjaalsinarey@gmail.com

**Keywords:** virtual reality exposure therapy, aged, 80 and over, exergames, postural balance, healthy aging, physical therapy, rehabilitation, virtual reality, accidental falls

## Abstract

Physical exercise has been recognized as an important strategy in the promotion of healthy aging. Positive effects on older adults’ motor ability are brought about by engaging their motor skills and promoting sensorimotor learning and cortical plasticity. These processes could be increased with the use of immersive virtual reality (IVR) technology, since the multisensory stimulation is greater. The aim of this study was to explore the usability and balance effects of an IVR exercise program in community-dwelling nonagenarian people. A sample of 12 women were allocated to an experimental group (EG *n* = 6; 91.67 ± 1.63 years) and a control group (CG *n* = 6; 90.83 ± 2.64 years). For 10 weeks, the EG used a commercial IVR exergame three times a week. All the sample completed the program without adverse effects (without Simulator Sickness Questionnaire symptoms). Post-gaming usability was good (System Usability Scale 78.33). The EG improved some balance parameters significantly (Tinetti test: balance (10.97 %; Sig = 0.017), gait (9.23%; Sig = 0.047) and total score (10.20%; Sig = 0.014) and maintained total TUG test times (−0.45%)). There were significant differences between groups (Tinetti test: balance (Sig = 0.004) and total score (Sig = 0.0032)). We successfully demonstrated that IVR training is feasible and is an effective and personalized method to enhance balance and to reduce the risk of falls in community-dwelling nonagenarian women.

## 1. Introduction

The main priority in the successful management of aging is enabling older people to be healthy, active, and autonomous for as long as possible [1]. Accordingly, the management of functional decline is an important issue [2]. Physical exercise has been recognized as an important health strategy in the promotion of healthy aging, since it can enhance the functioning of older adults who are already characterized as aging normally [3,4] The maintenance of physical functioning and independence is a key attribute of successful aging [5].

The current generation has higher rates of chronic disease and disability than any other [6]. Studies have shown that the four most common poor health conditions seen in older adults are decreased motor ability, increased obesity, impaired cognition and psychological disorders, which lead to a lower quality of life [7,8]. Due to aging, older adults exhibit a loss of muscle strength and balance [7], and, due to the deterioration of motor abilities, older adults’ risk of falls and fractures increases [6].

Falls are a leading cause of hospitalization due to injuries [9] and result in immense healthcare expenditure [10]. Older fallers suffering from balance problems constitute one of the most challenging types of patients in clinical practice [11]. Declines in strength (explosive and maximal) and balance (static and dynamic) performance have been reported to contribute to an increased risk of falling [12]. Cochrane reviews have concluded that exercise has a statistically significant beneficial effect on balance in the short term [13] and that regular neuromuscular exercise in the context of fall prevention can result in a meaningful reduction in fall events [14].

In comparison with older people of younger age, nonagenarians tend to participate in lower levels of physical activity, which leads to poorer functional independence [15]. They are the fastest-growing segment of the population in developed countries [16], so the performance of physical exercise is especially important in this age group [17].

One intervention strategy which has shown promise for promoting healthy aging among older adults is virtual reality (VR)-integrated exercise [18,19]. VR exercise is a novel and innovative technology, which immerses individuals in a computer-generated, multi-sensory, three-dimensional world wherein they interact with the virtual environment using a headset and/or exercise equipment [20,21]. VR technology can be characterized according to the degree of immersion (i.e., immersive or non-immersive). Immersive virtual reality (IVR) typically requires the use of a head-mounted display (e.g., Oculus Rift, Menlo Park, CA, USA) or an entire room display which encloses the user (e.g., the cave automatic virtual environment (CAVE)) [22]. Non-immersive VR, on the other hand, uses a computer-generated world which typically employs a desktop computer or projector [20]. Examples of non-immersive VR include the Nintendo Wii and the Xbox 360 Kinect [23].

As a consequence of aging, older adults naturally exhibit decreased motor ability, including compromised coordination, balance, muscular strength, and speed [23]. In general, VR exercise has demonstrated positive effects on these components of older adults’ motor ability by engaging older adults’ motor skills and promoting sensorimotor learning and cortical plasticity to improve motor ability [22]. These processes can be increased with IVR technology, since multisensory stimulation is greater and can enable better sensorimotor integration of the proposed tasks [24]. Several systematic reviews have shown the potential of VR tools to improve physical and functional abilities in older people, including strength, gait and balance [25,26,27,28,29].

Nonetheless, scientific evidence directly investigating the effects of exercise programs in nonagenarians is scarce [30]. Applications of exergame-based exercise programs with IVR targeting older adults remain relatively unexplored [22] and, to the best of our knowledge, have never been investigated in nonagenarians. This gap opens the door to research in this field.

Therefore, the aim of this study was to explore the usability and balance effects of an IVR personalized exercise program in nonagenarian older adults.

## 2. Materials and Methods

### 2.1. Participants

Participants were recruited from a community of elderly residents of an old people’s home in Pontevedra (Spain) and were invited to participate if they were be able to stand up and walk without aids, were aged ≥90 years, and had signed an informed consent document.

Participant exclusion criteria were the inability to correctly respond to the assessment protocol according to the clinician’s judgment; the presence of cardiovascular, pulmonary or musculoskeletal conditions that, according to a physiotherapist´s judgment, affected the patients’ ability to participate in the study; the presence of severe visual loss that could interfere with the ability to see the IVR simulation; and, finally, the presence of vertigo, epilepsy or psychosis.

A total of 12 subjects participated in this study (Table 1) who were then randomly allocated either to the experimental group (EG; *n* = 6) or to the control group (CG; *n* = 6).

The participants were all women, aged 91.67 ± 1.63 years (EG) and 90.83 ± 2.64 years (CG). All nonagenarians completed a Cognitive Mini-Exam without indication of cognitive impairment (MEC ≥ 24). All the experimental participants signed consent forms. The protocol conformed to the declaration of Helsinki and was approved by the Ethics Committee of the Servizo Galego de Saúde -SERGAS- (Spain) (code 2020/078).

### 2.2. Intervention

The EG performed a total of 30 IVR sessions of an exergame over a period of ten weeks. Three sessions were held every week and each IVR training session lasted for 6 min. At the same time, both the CG and the EG participated in the usual therapy programs of the center (45 min group sessions; five sessions per week), including occupational therapy involving activities related to general mobility and stretching, grooming, dressing, washing or using the phone or computer and managing their finances or daily medication, as well as cognitive stimulation workshops including cognitive exercises focused on memory, attention, language or calculation and strategies for managing stress, anxiety or depression.

**Figure 1:**
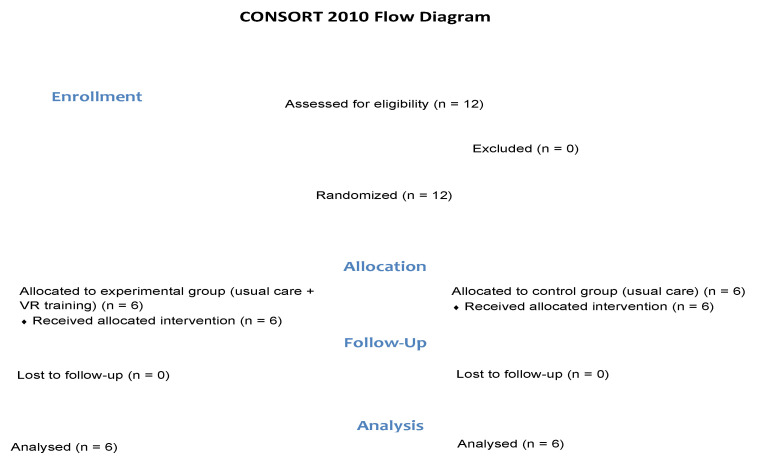
Study design: CONSORT 2010 flow diagram.

**Figure 2:**
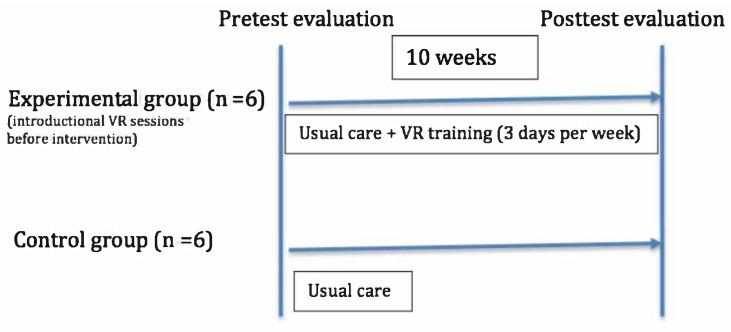
Study design: evaluation and timeline.

The study design is summarized in Figure 1 and Figure 2.

The immersive virtual environment was created using the HTC Vive Pro^TM^ commercial entertainment device. This system consists of a head-mounted display (HMD), two handheld controllers, two external sensors to delimit the gaming area, a wireless adapter and the Viveport software support (https://viveport.com accessed on 10 July 2021), supported by a desktop computer (CPU: Intel Core I7 7700 at 3.6 GHz, 1 TB HDD Sata 3.5 and NVIDIA GeForce RTX 2070 GPUs). A LED monitor was used to guide activities and set up the technical aspects of the device. A 6 m^2^ play area was defined following the manufacturer’s installation recommendations and taking into consideration the dimensions of the area selected for the study (the home´s gym room).

One week before the trial, the researchers, the home´s therapists and the participant sample had a meeting to facilitate an introduction to the IVR. The participants attending learned about the device and the controllers with an instructional talk and explanation of how to handle the device, both by demonstration and by actively trying out the controls and the HMD to take part in an immersive scenario (10 min approx.) where they could experience various games (mountain landscape or seabed scenarios), and receive instructions on handling.

**Figure 3:**
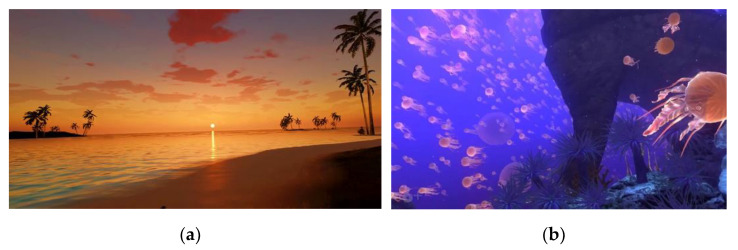
Screenshots of some immersive virtual scenarios proposed for first contact with IVR. (**a**) Sunset environment placed in a beach; (**b**) Seabed with Jellyfish.

During this time, the therapists asked the participants about the characteristics of their virtual environment, in order to test their involvement and immersion in the game. The subjects also performed body movements (real and virtual) and manipulated objects. This VR experience was offered to provide a first contact with the IVR in a pleasant, fun and quiet virtual scenario, in order to ensure good acceptability (Figure 3). At the end of the session, in a sitting position, the participants were asked if they had experienced any discomfort of the kind associated with cybersickness.

**Figure 4:**
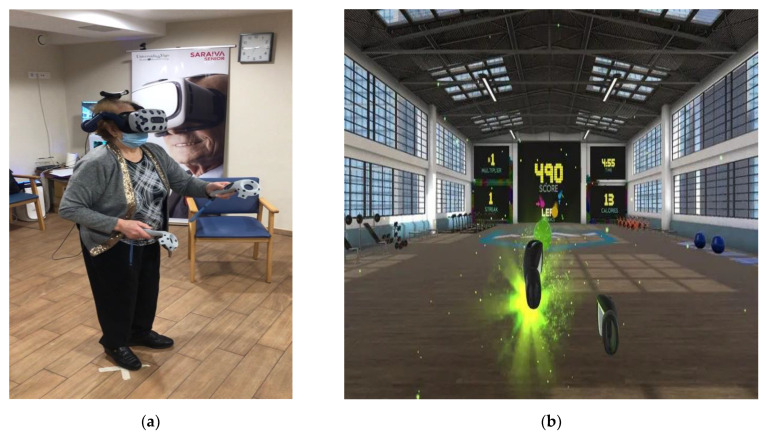
Real gym and virtual gym: (**a**) Participant during an exergame session; (**b**) Screenshot of the exergame proposed (BOXVR).

Based on our previous experiences [31,32], the clinical criteria of four physiotherapists, and by following general recommendations for physical activities for older adults [33], a BOX VR was chosen (available in the library of Viveport.com accessed on 10 July 2021), which simulates being in a gym. The participants must perform different boxing techniques (guard, jab, cross, hook, uppercut) and must move their trunk, head, and lower limbs, to vary their position if required, as well as performing coordinated movements and cardiovascular exercise. This game was chosen because it requires considerable body movement (joint mobility, muscle power, muscle tone) and provides a different form of physical interaction, very similar to the activities of a traditional physiotherapy session in older adults, and with a low probability of causing cybersickness symptoms (Figure 4). In previous studies, we have shown that virtual scenarios without accelerations or sudden changes of view minimize the impact of cybersickness [31,32]. Nevertheless, each participant undertook individual guided and supervised sessions, although all the subjects were present in the gym at the same time watching their peers train.

### 2.3. Assessments

The information collected included socio-demographic variables, such as age, sex, height, weight and body mass index (BMI), and the number of exergaming sessions completed. This data was acquired through interviews by trained researchers.

Balance parameters were obtained using the Tinetti test [34], and the Timed Up and Go test (TUG) [35] measured before and after 10 weeks of intervention. Participant experiences and the usability of IVR sessions were evaluated with the Simulator Sickness Questionnaire (SSQ, adapted and translated into Spanish) [36] and the System Usability Scale (SUS) [37] at the end of the VR-training.

The Tinetti test was developed by Tinetti et al. [34] to assess the gait and balance in older adults and their risk of falling. It comprises a gait subscale and a balance subscale. The maximum possible total score is 28, 12 for the gait subscale and 16 for the balance subscale. The Tinetti score subdivides patients into three groups depending on fall risk level: higher risk (≤18 points), moderate risk (19–23 points) and minimal risk (≥24 points).

The TUG test was created and validated by Podsiadlo and Richardson [35] to assess functional mobility in elderly persons. The test procedure consists of standing up from a chair, walking a distance of 3 m, turning around, returning to the chair and sitting down. The time needed to complete the predetermined route is measured with a stopwatch in seconds. Usually, the task is performed twice. Shorter times indicate better performance, and, in this case, were assessed with a Wiva® device placed on the L4–L5 spinal segment. (Letsense Group, Bologna, Italy). The Wiva® is an inertial detection device sensor which includes an accelerometer, a magnetometer and a gyroscope, and allows information to be recorded at the same time as the task is completed.

The SSQ, originally designed to be applied in simulators, consists of 16 items, grouped into three subscales and divided by symptomatology. Each item is assessed on a four-point scale (0 = I feel nothing, 1 = a little, 2 = medium, and 3 = a lot), and the total score results from the sum of the scores of the three subscales. This tool has been widely used to measure the frequency of cybersickness in the general population [38,39,40].

The SUS was developed as a survey that allows professionals to evaluate the usability of a product/service in a quick and easy manner. The SUS is a Likert-type scale that includes 10 questions. Participants rate each question from 1 to 5 according to their degree of agreement or disagreement with what they are reading, where 5 means that they completely agree and 1 means that they completely disagree. The ratings are combined using an algorithm providing a score out of a maximum of 100 points [41].

These assessment tools have been used in the evaluation of previous experiences [32,42] and were intended to assess the feasibility of using exercise-based IVR in the elderly population.

Taking into account the lack of VR studies in this population, the SSQ was provided to each participant at the end of the first six sessions (during the first two weeks of the trial) to detect possible adverse events of immersion. As a control measure, during the exergaming task, the average heart rate was monitored using an Mi Smart Band 4 wristband and an Mi Fit version 4.0.14 app (Xiaomi, Haidian, Beijing, China).

### 2.4. Statistical Analysis

Descriptive statistics (mean, standard deviation) were used to represent the demographic characteristics of the sample. For normal distribution analysis, a sample Shapiro–Wilk test (*p* > 0.05) was used. Differences in the sample were calculated using a Wilcoxon test (intragroup; Z, sig). A Kruskal–Wallis test (intergroup; H, sig) was performed to compare the effects of the program between the groups and between the two time points. All the tests were performed using the Statistical Package for the Social Sciences (SPSS Inc., Chicago, IL, USA) for MAC version 26.0. The level of significance was set at <0.05.

## 3. Results

There was high adherence to the intervention program (100% of the sample completed the IVR sessions). No significant differences were observed at baseline between the groups. The main outcomes are summarized in Table 2. The scores for the Tinetti test (gait, balance and total score) and the times for the TUG test (sit to stand, gait, turning, gait return, stand to sit and total time) in the EG and in the CG (pre- and post-test) are presented in Table 2.

The EG exhibited significantly improved Tinetti scores for balance (10.97 %; Sig = 0.017), gait (9.23%; Sig = 0.047) and total score (10.20%; Sig = 0.014). Overall, the EG maintained the total times for the TUG test (−0.45%); however, in the turning section, the times increased significantly after the intervention (−30.66%; Sig = 0.039).

The Tinetti scores worsened in the CG for balance (−15.18%; Sig = 0.020), gait (−1.84%) and total score (−9.30%). For the CG, the total times for the TUG test generally increased (−14.78%) after the intervention, with times for the turning section worsening in particular (−43.55%; Sig = 0.031). There were statistically significant differences between the groups in the Tinetti test scores for balance (Sig = 0.004) and total score (Sig = 0.032).

In relation to the experiences with the IVR sessions, Table 3 shows the usability and cybersickness values for the experimental group after the intervention. The reported usability was good (78.33/100) and no symptoms were recorded for the SSQ, either in the initial assessment period (the first six sessions), or in the post-test session.

## 4. Discussion

The primary outcome of this study was to explore the usability and balance effects of an IVR exercise program for nonagenarian older adults with a commercial HMD. Our findings show that a 10-week IVR protocol was feasible for nonagenarian women, without adverse effects (no SSQ symptoms), and with maximum adherence (no dropouts) and good usability. These results are consistent with the current literature, suggesting that IVR exposure is a positive option (in terms of safety and tolerability) for the promotion of physical activity in older adults [22] even though, in our pilot study, the population involved were community-dwelling nonagenarians, usually frailer people.

Although there are several studies that have employed exercise programs [30] in nonagenarian older adults, to the best of our knowledge, this is the first trial to apply IVR as an exercise facilitation tool in this age group. Our entire sample was composed of women, an imbalance which is to some extent evident in the epidemiological data, where the balance is tilted towards females for older ages [43]. Data from 2020 show that, globally, the number of women is slightly more than double that of men in the 90–94 age group (11,352,000 vs. 5,328,000), a proportion that increases in the 95–99 and 100+ age groups [44]. This data coincides with data for the region where the study was conducted: in Pontevedra there were 10,221 women and 4158 men aged 90 and over as of 1 July 2021 [45]. In the growing elderly population, women outnumber men and have distinct and differing sociodemographic and health characteristics. In old age, the simple fact of being a woman is a factor that generates significant inequalities; this study emphasizes that active aging should be a health objective for people of all ages and genders, based on optimization and equality of opportunity [46].

Exercises aimed at improving balance are essential in the elderly, mainly because they reduce the risk of falling [47]. The exergame investigated seeks to work on the general mobility of the body, but specifically to focus on the coordinated movements of the upper limbs and on quick reactions involving the trunk and lower limbs. The mobility entailed generates instabilities, variations in the center of gravity and weight transfers in the participant.

In our study, the EG showed a significant improvement in the Tinetti scores, particularly in the balance section; in addition, there were significant differences between both groups in favor of the EG. TUG times showed similar values in the CG and a slight increase in the EG. It is possible that the tasks in exergaming differ significantly from activities such as “turns” or “stand to sit” (items where there was a greater increase in TUG times). Nevertheless, from our point of view, the maintenance of functional capacities in this population can be considered a good indicator (the EG maintained total TUG times), and a virtual exercise program has been shown to be as feasible as other programs aimed at nonagenarians [48] where only conventional exercise training was used.

The Tinetti test provides an indicator of the risk of falls. Considering that, at baseline, both groups presented a moderate risk of falls (19–23; 22 GE and 21.5 GC), it is noteworthy that the EG had a mean score of 24.5 in the post-test, so that application of the IVR program resulted in EG participants being successfully placed in the minimum risk stratum (≥24). On the other hand, although the results for the CG worsened for the Tinetti test, the obtained score remained in the moderate risk stratum, suggesting that the programs usually carried out at the center had been able to slow down progression in the loss of functional capacities.

Early insights about IVR have been used to help rehabilitate subjects suffering from stroke and multiple sclerosis [49], cognitive impairment [50], and Parkinson´s disease [51]. However, the use of this technology has been limited in fall prevention and our study opens the door to future research in the field of virtual reality in nonagenarians and for other age-related conditions. The scores obtained on usability of the device and the exergame were in line with those found in previous studies [32]. In addition, some ratings in SUS responses such as “I felt very confident using the system” (4/4), “I found the various functions in this system were well integrated” (4/4), and “I thought the system was easy to use” (3.83/4) reinforce the suitability of the proposal.

### 4.1. Limitations

There were some limitations to this study. First, to better generalize our results, future studies including a larger sample size that is more representative of the nonagenarian population are needed. Furthermore, our participants were women (100% of the sample), and future studies regarding gender differences on the effect of the IVR intervention are needed. Another limitation is the possible brevity of the intervention, as we do not know if the values found could be improved over longer interventions, something that could have influenced the lack of significant results in the TUG test. Finally, the lack of a follow-up in the period after the intervention was completed makes it impossible to know the permanence of improvements achieved in the Tinetti test, even if usual care is maintained.

### 4.2. Implications of Using Immersive Virtual Reality in Clinical Practice

Although the incorporation of immersive systems into daily clinical practice requires more evidence, our study shows that physical rehabilitation can be supported by exercise-based training applications for which the use of IVR promises increased motivational and training effects. The use of IVR can ensure higher levels of adherence to therapies. It can also make available a large amount of material resources, in virtual mode, which would otherwise be impossible for physiotherapists to use in real environments. In turn, IVR offers multiple options related to specific therapeutic strategies for each target population.

## 5. Conclusions

In conclusion, we have successfully demonstrated that IVR training, with a commercial HMD and the exergame proposed, is a feasible and effective method to enhance balance and reduce the risk of falls in female nonagenarian old people´s home residents. The IVR training outlined in this study could complement other fall prevention intervention programs. The exergame proposed here offers a safe and well-accepted intervention with high levels of effectiveness and adherence, which could provide successful personalized treatment for the prevention of falls in community-dwelling older adults in the near future.

## Figures and Tables

**Table 1:** Characteristics of the sample.

	Experimental Group (*n* = 6)		Control Group (*n* = 6)	
	Mean	SD	Mean	SD
Age (years)	91.67	1.63	90.83	2.64
Height (m)	1.51	0.03	1.45	0.07
Weight (kg)	61.65	14.32	54.87	9.87
MEC	27.66	2.87	24.45	1.56
BMI (kg/m^2^)	26.95	6.02	25.95	3.45

BMI, Body Mass Index. SD, Standard Deviation. MEC, Cognitive Mini-Exam.

**Table 2:** Balance scores (static and dynamic metrics) in the sample.

		Experimental Group (*n* = 6)						Control Group (*n* = 6)						Anova (2 × 2)
		Pre		Post		% Improve	Wilcoxon Test	Pre		Post		% Improve	Wilcoxon Test	
		Mean	SD	Mean	SD			Mean	SD	Mean	SD			
Tinettii test	Balance	12.17	1.60	13.67	1.03	10.97	Z = −3.503; **Sig = 0.017**	12.67	1.03	11.00	0.89	−15.18	Z = 3.371; **Sig = 0.020**	H_1,24_ = 10.939; **Sig = 0.004**
	Walk	9.83	1.94	10.83	0.98	9.23	Z = −2.236; **Sig = 0.047**	8.83	2.14	8.67	1.37	−1.84	Z = −0.349; Sig = 0.741	H_1,24_ = 0.731; Sig = 0.403
	Total	22.00	2.45	24.50	1.52	10.20	Z = −3.727; **Sig = 0.014**	21.50	3.08	19.67	1.86	−9.30	Z = 2.101; Sig = 0.090	H_1,24_ = 5.298; **Sig = 0.032**
Timed up and go test (TUG)	Sit to Stand	2.60	1.36	3.49	2.76	−25.50	Z = −1.376; Sig = 0.227	5.99	1.94	7.19	4.26	16.68	Z = −0.755; Sig = 0.484	H_1,24_ = 0.018; Sig = 0.894
	Gait	5.73	3.45	5.04	0.79	13.69	Z = 0.505; Sig = 0.635	7.47	3.48	7.42	3.61	0.67	Z = 0.023; Sig = 0.983	H_1,24_ = 0.066; Sig = 0.801
	Turning	1.56	0.51	2.25	1.02	−30.66	Z = −2.023; **Sig = 0.039**	2.41	0.78	4.27	5.07	−43.55	Z = −2.005; Sig = 0.031	H_1,24_ = 0.294; Sig = 0.594
	Gait return	5.58	3.73	5.07	3.59	10.05	Z = 1.055; Sig = 0.340	6.46	2.99	6.73	1.78	−4.01	Z = −0.302; Sig = 0.775	H_1,24_ = 0.093; Sig = 0.764
	Stand to sit	4.10	1.24	5.27	2.06	−22.20	Z = −2.236; Sig = 0.144	5.34	1.73	6.36	2.60	−16.03	Z = −0.930; Sig = 0.395	H_1,24_ = 0.008; Sig = 0.931
	Total	19.56	8.95	19.65	8.94	−0.45	Z = −2.236; Sig = 0.917	27.67	8.33	32.47	10.42	−14.78	Z = −2.198; Sig = 0.079	H_1,24_ = 0.393; Sig = 0.538

**Table 3:** Cybersickness and usability in the experimental group.

	Mean	SD
**Simulation Sickness Questionnaire (SSQ)**		
Total Score	0.00	0.00
**System Usability Scale (SUS)**		
1. I think that I would like to use this system frequently.	3.00	1.41
2. I found the system unnecessarily complex.	2.50	1.22
3. I thought the system was easy to use.	3.83	0.98
4. I think that I would need the support of a technical person to be able to use this system.	2.00	1.67
5. I found the various functions in this system were well integrated.	4.00	0.63
6. I thought there was too much inconsistency in this system.	3.33	1.21
7. I would imagine that most people would learn to use this system very quickly.	3.17	0.75
8. I found the system very cumbersome to use.	2.83	1.47
9. I felt very confident using the system.	4.00	0.63
10. I needed to learn a lot of things before I could get going with this system.	3.33	0.82
Total Score	78.33	7.69

## Data Availability

The data presented in this study are available on request from the corresponding author.
